# Screening for Streptomycin Resistance-Conferring Mutations in *Mycobacterium tuberculosis* Clinical Isolates from Poland

**DOI:** 10.1371/journal.pone.0100078

**Published:** 2014-06-17

**Authors:** Tomasz Jagielski, Helena Ignatowska, Zofia Bakuła, Łukasz Dziewit, Agnieszka Napiórkowska, Ewa Augustynowicz-Kopeć, Zofia Zwolska, Jacek Bielecki

**Affiliations:** 1 Department of Applied Microbiology, Institute of Microbiology, Faculty of Biology, University of Warsaw, Warsaw, Poland; 2 Deparment of Bacterial Genetics, Institute of Microbiology, Faculty of Biology, University of Warsaw, Warsaw, Poland; 3 Department of Microbiology, National Tuberculosis and Lung Diseases Research Institute, Warsaw, Poland; St. Petersburg Pasteur Institute, Russian Federation

## Abstract

Currently, mutations in three genes, namely *rrs*, *rpsL*, and *gidB*, encoding 16S rRNA, ribosomal protein S12, and 16S rRNA-specific methyltransferase, respectively, are considered to be involved in conferring resistance to streptomycin (STR) in *Mycobacterium tuberculosis*. The aim of this study was to investigate the spectrum and frequency of these mutations in *M. tuberculosis* clinical isolates, both resistant and susceptible to STR. Sixty-four *M. tuberculosis* isolates recovered from as many TB patients from Poland in 2004 were included in the study. Within the sample were 50 multidrug-resistant (32 STR-resistant and 18 STR-susceptible) and 14 pan-susceptible isolates. Preliminary testing for STR resistance was performed with the 1% proportion method. The MICs of STR were determined by the Etest method. Mutation profiling was carried out by amplifying and sequencing the entire *rrs*, *rpsL*, and *gidB* genes. Non-synonymous mutations in either *rrs* or *rpsL* gene were detected in 23 (71.9%) of the STR-resistant and none of the STR-susceptible isolates. Mutations in the *gidB* gene were distributed among 12 (37.5%) STR-resistant and 13 (40.6%) STR-susceptible isolates. Four (12.5%) STR-resistant isolates were wild-type at all three *loci* examined. None of the *rrs*, *rpsL* or *gidB* mutations could be linked to low, intermediate or high level of STR resistance. In accordance with previous findings, the *gidB* 47T→G (L16R) mutation was associated with the Latin American-Mediterranean genotype family, whereas 276A→C (E92D) and 615A→G (A205A) mutations of the *gidB* gene were associated with the Beijing lineage. The study underlines the usefulness of *rrs* and *rpsL* mutations as molecular markers for STR resistance yet not indicative of its level. The *gidB* polymorphisms can serve as phylogenetic markers.

## Introduction

Streptomycin (STR), an aminocyclitol glycoside antibiotic, was the first drug used successfully for the treatment of tuberculosis (TB) in the 1940s. Since then, STR has become the drug of first choice for all forms of TB, and, after the introduction of isoniazid (INH), rifampicin (RMP), pyrazinamide (PZA), ethambutol (EMB), and other anti-TB drugs, an important component of combination therapies for the disease. However, the high rates of STR resistance, primarily due to the historical use of the drug in a monotherapy application, the risk of toxicity, including serious neurotoxic reactions, the parenteral route of administration, and, above all, the lower efficacy relative to the succeeding drugs, lead to a gradual decline in the use of STR. Under the current WHO guidelines, the standard treatment regimen for drug-susceptible TB excludes STR and comprises a combination of INH, RMP, PZA, and EMB during the intensive phase of treatment, followed by INH together with RMP in the continuation phase [1]. Nevertheless, STR continues to be an integral part of chemotherapy for TB. Not only is the drug a component of the retreatment regimens, but also, in many parts of the world, it is among the first-line agents to be administered in drug-resistant TB (DR-TB) cases, yet susceptible to aminoglycosides [1].

The mechanism of action of STR in mycobacteria, largely inferred from the research on *Escherichia coli*, involves irreversible binding to the ribosomal protein S12 and 16S rRNA, which are the constituents of the 30S subunit of the bacterial ribosome. Through this interaction, STR interferes with translational proofreading and thereby inhibits protein synthesis [2].

Resistance of tubercle bacilli to STR appears to be mediated by mutations in the *rpsL* and *rrs* genes, encoding the ribosomal protein S12 and the 16S rRNA, respectively. Amino acid substitutions in the S12 protein affect STR binding by perturbing the higher-order structure of 16S rRNA. Alterations in the 16S rRNA itself result in a severely decreased affinity for STR [2].

Furthermore, mutations in the *rpsL* gene have been associated with a high-level of STR resistance, whereas mutations in the *rrs* gene have been shown to confer an intermediate-level of resistance [3]–[7]. Quite recently, mutations in the *gidB* gene, coding for a 7-methylguanosine methyltransferase (m7G) specific for the 16S rRNA, have been implicated in STR resistance in *M. tuberculosis*. In addition, the *gidB* polymorphisms have been correlated with a low-level of STR resistance [8]–[10].

The rapid determination of drug resistance patterns of *M. tuberculosis* clinical isolates is an essential prerequisite for the prompt implementation of effective chemotherapy and early interruption of DR-TB transmission. Currently, screening for mutations in genetic *loci* associated with drug resistance is the most promising approach for rapid and reliable diagnosis of DR-TB. In case of STR, the association between specific mutations in the two aforesaid genes (*rpsL* and *rrs*) and drug-resistant phenotype, and, consequently, the utility of these mutations as highly-specific predictive markers of STR resistance has been well recognized. Since the type and frequency of STR resistance mutations vary depending on the population and geographical area studied, it is imperative to establish the global prevalence of each particular mutation, before it can be used as an accurate predictor for resistance to this drug.

The purpose of this study was to investigate the spectrum and frequency of mutations in the *rpsL*, *rrs*, and *gidB* genes in STR-resistant and STR-susceptible *M. tuberculosis* clinical isolates from Poland.

## Materials and Methods

### Strains

Sixty-four clinical isolates of *M. tuberculosis*, deposited in the culture collection of the National TB and Lung Diseases Research Institute in Warsaw, were used for the study. Included in that number were 50 multidrug-resistant (MDR) (32 STR-resistant and 18 STR-susceptible) and 14 pan-susceptible isolates. The isolates were obtained from pulmonary TB patients diagnosed in different parts of Poland and collected during the third national survey of DR-TB (i.e. from 1 January to 31 December 2004). The study sample included all MDR-TB cases collected in the survey. Primary isolation, cultivation, and species identification were done by using standard mycobacteriological methods, essentially described elsewhere [11].

### Drug Susceptibility Testing

The first-line drug susceptibility testing (DST) was performed with the conventional proportion method on Löwenstein-Jensen (L–J) medium, strictly according to the WHO guidelines [12]. The following critical drug concentrations were used: 0.2 µg/ml (INH), 40 µg/ml (RMP), 4 µg/ml (STR), and 2 µg/ml (EMB). Resistance was defined by the growth of ≥1% of a bacillary inoculum on drug-containing medium compared to the drug free control.

The minimal inhibitory concentrations (MICs) of STR were determined by the Etest method (bioMérieux, Marcy-l’Étoile, France), following the instructions of the Etest technical guide, provided by the manufacturer, and as described previously [13]. Briefly, colonies from 4-week-old cultures on L-J slants were suspended in Middlebrook 7H9 broth and adjusted to a turbidity equivalent of a 3.0 McFarland standard. The 90-mm plates containing Middlebrook 7H11 agar with 10% oleic acid albumin dextrose complex (OACD) were swabbed evenly with the inocula to ensure uniform growth. After a 24-hour incubation at 37°C in 5% CO_2_, Etest stripes, with a gradient of STR ranging from 0.016 to 256 µg/ml, were applied onto the surface and the plates were reincubated as described above for 3 weeks or more until the ellipse of inhibition was clearly visible. The MICs were read at the point where the inhibition ellipse intersected the scale on the Etest strip. The breakpoint MICs for the Etest method were the same as those used for the proportional method. To validate the performance of the DST, including the Etest assay, the reference strain *M. tuberculosis* H37Rv, susceptible to all the drugs tested, was used as the quality control strain. All isolates were tested in duplicate to assure the accuracy of the results.

The use of the Etest method to determine the MICs of STR was supported by previous studies showing an overall high agreement with the traditional techniques, such as the proportion method in L-J or Middlebrook agar media [13], [14].

### DNA Extraction

Genomic DNA was extracted from *M. tuberculosis* cultures on L-J slants by the lysozyme/proteinase K cetyl-trimethyl ammonium bromide (CTAB) method [15]. The purified DNA was dissolved in TE buffer (10 mM Tris-HCl, 1 mM EDTA, pH 8.0) and quantified with the NanoDrop ND-1000 Spectrophotometer (NanoDrop Technologies, USA). The DNA samples were stored at –20°C until used.

### PCR Amplification and Sequence Analysis

The complete open reading frames of the *rpsL*, *rrs*, and *gidB* genes were subjected to mutational analysis by direct sequencing. The oligonucleotide primers (Table 1) used for PCR amplification of the three genes were newly designed in this study, based on the sequences of the respective genes of *M. tuberculosis* reference strain H37Rv, available at the TubercuList database (http://genolistpasteurfr/TubercuList/). The 50-µl PCR mixtures contained 25 µl of the HotStarTaq Master Mix (Qiagen), 0.1 µM of each primer, and 1 µl (*ca*. 20 ng) of template DNA. All PCRs were run on a T100 Thermal Cycler (Bio-Rad) under the following amplification conditions: initial denaturation at 95°C for 3 min, followed by 34 cycles of denaturation at 95°C for 30 s, annealing at 57°C (*rpsL*), 55°C (*gidB*) or 54°C (*rrs*) for 30 s, and extension at 72°C for 20 s (*rpsL*), 50 s (*gidB*) or 1 min and 10 s (*rrs*), with a final extension at 72°C for 10 min. The products of amplification were analyzed by electrophoresis in 1.5% agarose gels, purified with the Clean-Up kit (A&A Biotechnology) and sequenced by using the BigDye ver. 3.1 Terminator Cycle Sequencing Kit (Applied Biosystems) in the ABI 3130×l Genetic Analyzer (Applied Biosystems). In order to exclude any sequence ambiguities, all sequencing reactions were carried out in both forward and reverse directions using the same primer pairs as for the PCR amplifications and, only for the *rrs* gene, an additional pair of internal sequencing primers (Table 1). The sequence data were assembled and analyzed with the ChromasPro (ver. 1.7.1) software (Technelysium), and the resulting consensus sequences were aligned with the corresponding, wild-type sequences of the reference *M. tuberculosis* H37Rv strain (TubercuList; http://genolistpasteurfr/TubercuList/) using the BLASTN software (http://blast.ncbi.nlm.nih.gov/).

**Table 1:** Primers used for PCR amplification and sequencing.

Gene	Primer^a^		Product size [bp]
	**Designation**	**Nucleotide sequence (5′→3′)**	
*rpsL*	Fw: rpsLF	GGCATGGCCGACAAACAGAACG	501
	Rev: rpsLR	ACTGGGTGACCAACTGCGATCC	
*rrs*	Fw: rrsF	TGGCCATGCTCTTGATGC	1707
	Rev: rrsR	CGCCCACTACAGACAAGAAC	
	rrs-seqF*	TTTACGGCGTGGACTACC	
	rrs-seqR*	CAGTAACTGACGCTGAGGAG	
*gidB*	Fw: gidBF	GGAGTGCGTAATGTCTCC	785
	Rev: gidBR	GTCGGTGGTGTCATTTCC	

a Fw and Rev stand for forward and reverse (primers), respectively. All starters used for amplification were also used for sequencing. An asterisk (^*^) indicates additional intragenic starters used for sequencing.

### Nucleotide Sequence Accession Numbers

The nucleotide sequences of the *rpsL*, *rrs*, and *gidB* genes containing novel mutations were deposited in the GenBank database (National Center for Biotechnology Information; http://www.ncbi.nlm.nih.gov/) under the following accession numbers: KF740612, KF740621, and KF740622 for the *rpsL* gene mutants, KF796660 to KF796662, and KF796665 for the *rrs* gene mutants, and KF740589, KF740590, KF740598 to KF740601, KF740603 to KF740605, KF740607, KF740609, KF740611, KF796668, and KF796669 for the *gidB* gene mutants.

### Genotyping

All isolates were subjected to spoligotyping, essentially as described elsewhere [16]. Spoligotype signatures and phylogenetic (sub–)clades were assigned according to the SITVIT WEB database housed by the Institut Pasteur de Guadeloupe (http://www.pasteur-guadeloupe.fr:8081/SITVIT_ONLINE/). Clade assignment of the spoligotypes not found in SpolDB4 was done with SPOTCLUST, an algorithm based on the SpolDB3 database, described previously [17] and available online (http://cgi2.cs.rpi.edu/~bennek/SPOTCLUST.html).

### Statistical Analysis

The associations between mutations and MICs of STR and between mutations and spoligotyping families were assessed with the non-parametric Mann-Whitney U test, Kruskal-Wallis test and χ^2^ test. A *P* value of <0.05 was considered statistically significant. All analyses were performed using the SPSS ver. 22.0 (SPSS Inc., USA) statistical software package.

## Results

Among the 32 *M. tuberculosis* isolates examined, defined as MDR and STR-resistant by the proportion method, 27 (84.4%) were also identified as STR-resistant by the Etest methodology. For these isolates, the MICs of STR were within the range of 64 to >1024 µg/ml. For the other half of the sample, comprising 18 MDR isolates susceptible to STR and 14 pan-susceptible isolates, the Etest STR MICs were between <0.064 and 1.5 µg/ml, falling into the susceptible category. The MIC_50_s for STR-resistant and susceptible isolates were 512 µg/ml and <0.064 µg/ml, respectively, whereas the MIC_90_s for these two groups of isolates were >1024 µg/ml and 1.5 µg/ml, respectively. Among the STR-susceptible isolates, the MIC_90_s were markedly lower for the pan-susceptible isolates than for the MDR isolates (<0.064 *vs* 1.5 µg/ml).

The overall concordance between the proportion method and Etest method for determining susceptibility of tubercle bacilli to STR was high and equaled 92.2% (only 5 out of 64 strains produced discordant results, being STR-resistant by the proportion method, while STR-susceptible by the Etest method).

**Figure 1:**
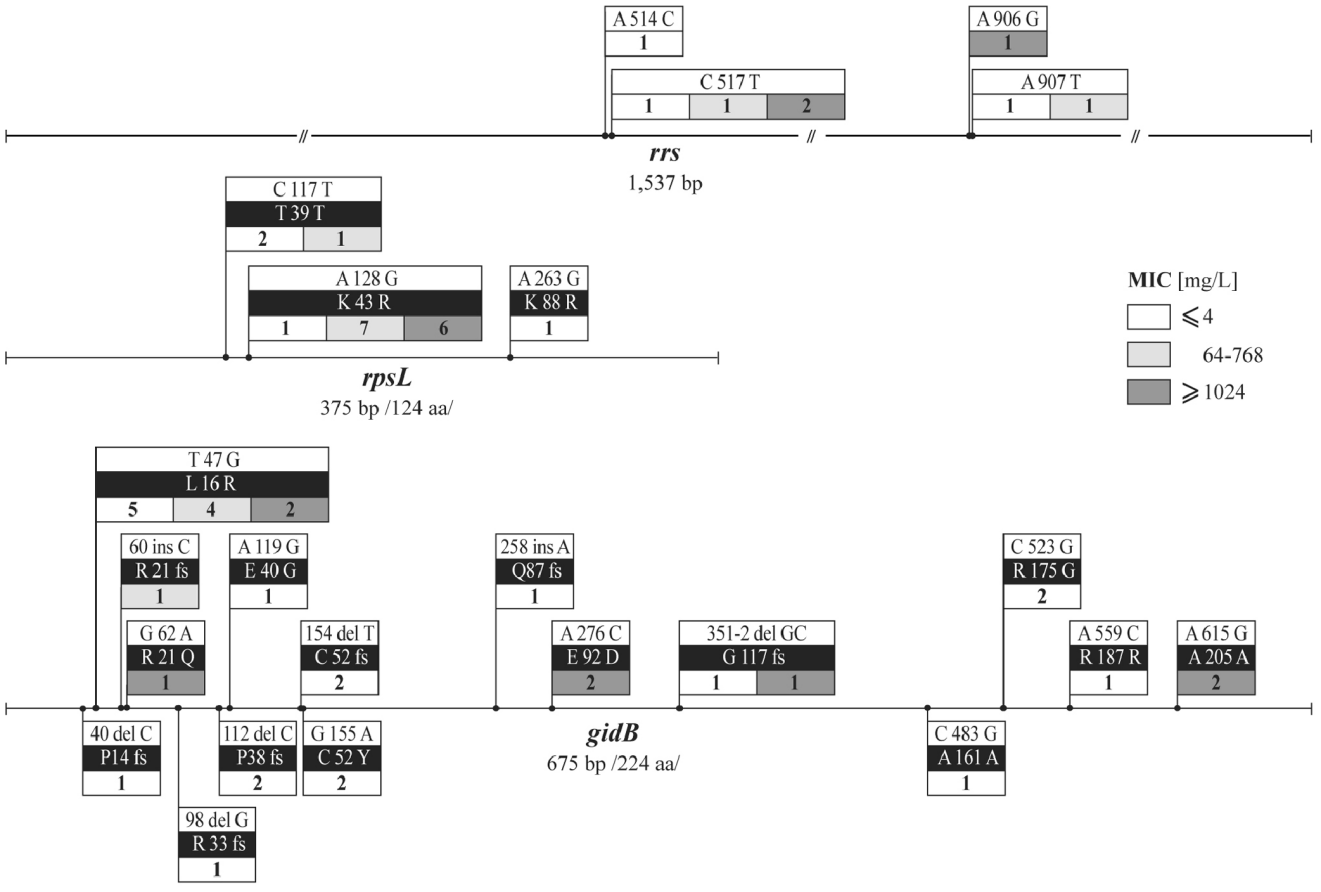
Schematic representation of the distribution of mutations in the *rrs*, *rpsL*, and *gidB* gene, with regard to MIC values of STR. del, deletion; ins, insertion; fs, frameshift mutation. Numbers in white or grey-shaded boxes, below mutation designations, represent isolates with a particular mutation type and MIC of STR.

Both STR-resistant and STR-susceptible isolates were screened for mutations in the *rrs*, *rpsL*, and *gidB* genes (Figure 1). Mutations in the *rrs* gene were identified in 8 (25%) of the STR-resistant, and none of the STR-susceptible isolates. The *rrs* mutations were of four types. Four isolates had a C→T transition at nucleotide (nt) position 517. Two isolates had an A→T transversion at nt position 907. One isolate had a transversion A→C at nt position 514, and another one – a transition A→G at nt position 906. Two isolates with 517C→T or 907A→T mutation had their STR MICs below the resistance breakpoint value (0.25 and 0.75 µg/ml, respectively). For the remaining 6 *rrs* mutants, the MICs were ≥96 µg/ml.

Single mutations in the *rpsL* gene were detected in 16 (50%) and 2 (11.1%) of the MDR isolates that were resistant (MIC range: 0.75–>1024 µg/ml) and susceptible (MICs of 0.125 and 0.38 µg/ml) to STR, respectively. Of the former, all but two isolates had an A→G mutation at nt position 128, resulting in a K43R amino acid substitution. One isolate had a similar amino acid change (K88R), due to an A→G mutation at nt position 263. One isolate had a silent mutation (117C→T; T39T). This mutation was also present in two STR-susceptible isolates. Two isolates with a K43R or K88R mutation had their STR MICs below the resistance breakpoint value (3 and 0.75 µg/ml, respectively).

Mutations in the *rrs* and *rpsL* genes were mutually exclusive, that is isolates with *rrs* mutations had no *rpsL* mutations and vice versa.

Sixteen different mutation types in the *gidB* gene were identified, distributed among 23 (46%) MDR isolates (12 (37.5%) out of 32 STR-resistant and 11 (61.1%) out of 18 STR-susceptible) and 2 (14.3%) pan-susceptible isolates. Ten different types of *gidB* mutations were observed only in STR-susceptible isolates (9 MDR isolates and one pan-susceptible isolate; MIC range: <0.064–2 µg/ml). These mutations included 5 frameshift mutations due to a single nt deletion (112delC in 2 isolates, 154delT in 2 isolates, 40delC in one isolate, and 98delG in one isolate) or insertion (258insA in one isolate) and 5 substitution mutations: 119A→G (E40G), 155G→A (C52Y), 483C→G (A161A), 523C→G (R175G), and 559A→C (R187R), which all occurred in single isolates, except for 155G→A and 523C→G mutations which co-existed in 2 isolates. The 559A→C polymorphism was found in a pan-susceptible isolate.

Four *gidB* mutation types were found exclusively in STR-resistant isolates (MICs of 512 and >1024 µg/ml in one, and 3 isolates, respectively). Two different substitutions, i.e. 276A→C (E92D) and 615A→G (A205A) co-occurred in 2 isolates, whereas substitution 62G→A (R21Q) and a frameshift mutation (60insC) were found in single isolates. Two *gidB* mutation types were detected in 13 isolates, both STR-susceptible and STR-resistant. A substitution 47T→G (L16R) was present in 10 MDR isolates (8 STR-resistant and 2 STR-susceptible) and one pan-susceptible isolate, whereas a frameshift mutation caused by a two-nt deletion (del351-2GC) – in 2 MDR isolates, one with and the other without resistance to STR.

Of the 25 isolates with an altered *gidB* gene sequence, 17 (68%) had a single mutation, 6 (24%) had two, and 2 (8%) had three mutations simultaneously.

Ten (15.6%) of the STR-resistant isolates contained mutations in two *loci*, i.e. in *gidB* and *rrs* (2 isolates) or *gidB* and *rpsL* (8 isolates). Four (12.5%) STR-resistant isolates whose MICs were of 2, 512, and >1024 µg/ml had wild-type sequences at all three *loci* analyzed. In one STR-resistant (MIC of 512 µg/ml) isolate a silent mutation (117C→T; T39T) in the *rpsL* gene was the only mutation detected.

The MICs for STR did not differ between the *gidB* mutation-containing and *gidB* mutation-free isolates (*P*>0.05). Likewise, neither the number of mutations in the *gidB* gene nor the number of mutated *loci* were correlated with the MIC values for STR. However, the *gidB* mutation-containing isolates were considerably more susceptible to STR than the *rrs* or *rpsL* mutation-containing isolates. For these three groups of isolates, the MIC_50_s were of 1.5, 256, and 256 µg/ml, respectively (Table S1).

**Figure 2:**
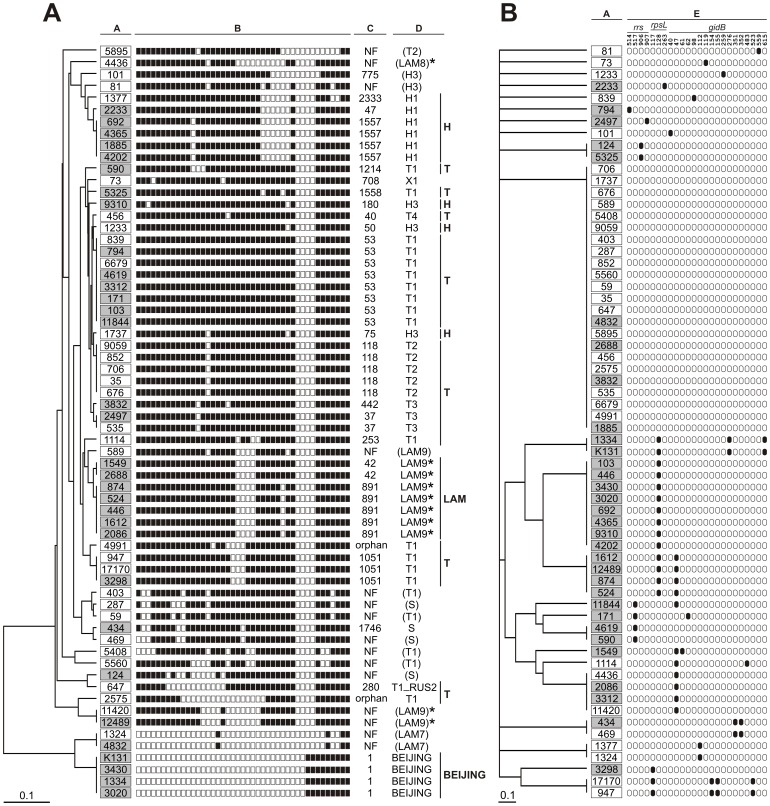
Dendrograms (DendroUPGMA, Jaccard model, UPGMA) based on spoligotype patterns (a) and mutation profiles at *rrs*, *rpsL*, and *gidB* genes (b) of the 64 *M. tuberculosis* clinical isolates under the study. A, isolate no.; B, spoligotype pattern (binary); C, spoligotype signature (shared international type, SIT); D, phylogenetic clade; E, mutation profile. Numbers representing isolates resistant or susceptible to STR were framed against grey and white backgrounds, respectively. The presence or absence of an individual nucleotide change was shown as black and white spots, respectively. NF, not found (in the SITVIT WEB database); orphan, spoligotype represented by a single isolate in the SITVIT WEB database. The SPOTCLUST-assigned clades are given in brackets. Asterisks indicate the confirmed assignment to the LAM lineage, based on the presence of LAM-specific mutations, i.e. 309G>A and 1212 C>G substitution, in *Rv0129c* and *Rv3062* genes, respectively [36], [37].

The results of mutation profiling at the three *loci* analysed (*rrs*, *rpsL*, and *gidB*) and the spoligotyping results of all the 64 *M. tuberculosis* isolates studied were shown in figure 2.

## Discussion

In *M. tuberculosis* resistance to STR is associated with alterations within the *rrs*, *rpsL*, and *gidB* genes whose products are involved in the 30S ribosomal subunit composition. Although a wide spectrum of distinct mutations in those genes has been characterized, there is still relatively little information on their frequency and distribution among STR-resistant and STR-susceptible isolates from geographically different localities, as well as on the relationship of the mutations with the level of STR resistance.

All *rrs* gene mutations found in this study localized in two highly mutable regions known as the 530 loop (514A→C; 517C→T) and the 912 loop (906A→G; 907A→T). All these mutations, except one (906A→G), have previously been reported in STR-resistant *M. tuberculosis* isolates, most of which were MDR [7], [18]–[20]. The 906A→G substitution is novel but other mutations in the same position (906A→C; 906A→T) have already been described [21]–[24]. According to the literature, neither of these four *rrs* mutation types were observed in isolates susceptible to STR. Mutations in the *rrs* gene accounted for a quarter (8/32) of the STR-resistant isolates, reaching the highest mutation frequencies for this gene reported, ranging from 0–28% [6], [18], [19], [22], [24]–[28]. Compared with the *rrs* gene, mutations in the *rpsL* gene was associated with twice as many STR-resistant isolates (16/32). Of the three mutation types identified for the *rpsL* gene, two (K43R and K88R) are the most common genetic alterations present in STR-resistant *M. tuberculosis* isolates. A single mutation K43R, representing 87.5% of the *rpsL* mutants and 43.7% of the STR-resistant sample from this work, ranks first in most of the studies characterizing the mutation profiles associated with STR resistance in tubercle bacilli. Yet, the detection rate for this mutation varies considerably between geographical areas, being of 13.2% in Mexico [26], 25% in Brazil [29], 42.9% in North India [30], 52.8% in Korea [18], 70.4% in China [22], and 80.4% in Singapore [31]. Of note is that the highest frequencies of the K43R substitution are in areas where strains of the Beijing genotype family predominate.

The K43R is also the leading mutation among those STR-resistant isolates that display an MDR phenotype, with the mutation frequency of 24–64.1% [18], [19], [29], [32], [33]. The high prevalence of the K43R mutation among STR-resistant *M. tuberculosis* isolates can be explained by the fact that this particular mutation carries no fitness cost for the bacteria [34]. The K88R substitution usually plays a minor role and occurs much less frequently than K43R. Only a very few studies have documented those two mutations equally represented among STR-resistant isolates [4]–[6], [27]. In the great majority of studies in which STR-susceptible isolates were also screened for mutations in the *rpsL* gene, neither codon 43 nor 88 was changed. However, mutations in these codons, including the commonest K→R amino acid replacements do occur in susceptible isolates, albeit very rarely [27], [35]. The only other mutation found in the *rpsL* gene was a silent mutation in codon 39 (T39T). This mutation, which had only once been described before this study, seems not to be associated with STR resistance, as it was observed in STR-resistant and STR-susceptible isolates.

Double mutants at both *rrs* and *rpsL* genes were absent from the sample analyzed. Mutations in both these *loci* have very rarely been observed to occur concurrently [4], [25], [26], [31]. This may suggest that mutational alterations in either *rrs* or *rpsL* may alleviate the need for the modification of the other gene.

An important observation from this study was that *rrs* and *rpsL* mutation-containing isolates did not differ from each other in terms of STR resistance level, as evidenced by comparing the MIC_50_, MIC_90_, and median MIC values for the two groups (256, >1024, and 384 µg/ml, accordingly). This is congruent with the study of Shi et al. [22] who found no relationship between mutation types in the *rrs* or *rpsL* genes and levels of STR resistance, but contrasts with several other studies in which mutations in the *rpsL* gene correlated with high-level resistance to STR (>250 µg/ml), whilst alterations in the *rrs* gene correlated with intermediate to low drug resistance level (<50 µg/ml) [3], [4], [6], [7]. One reason for this discrepancy may relate to the nature of the sample studied, which included MDR-TB isolates. Therefore, STR resistance, if occurred, was always compounded with MDR phenotype.

Sixteen different types of *gidB* mutations, including nine novel (not reported so far) types, were identified in the sample studied. This is consistent with the highly polymorphic nature of the *gidB* gene observed by other authors. For instance, Feuerriegel et al. [27] reported 16 *gidB* mutations among 56 *M. tuberculosis* isolates, whereas Spies et al. [29] detected 18 mutations in only 19 isolates. In both these studies, as in others, *gidB* mutations occurred in both STR-susceptible and -resistant isolates [7], [9], [10], [18]–[20], [27], [29]. This was also the case in the present study. Twelve *gidB* mutation types were found in STR-susceptible isolates. The remaining four types were detected only in resistant isolates. However, two of the latter mutations, a deletion mutation (98delG) and a silent mutation (615A→G; A205A), have previously been reported as occurring also in susceptible isolates, and thus cannot be considered as related to STR resistance [7], [18]–[20], [27]. Only two newly described mutations, a substitution 62G→A (R21Q) and a frameshift mutation 60insC, might be involved in STR resistance, and in particular the latter, which, apart from being absent in susceptible isolates, did not coincide with any *rrs* or *rpsL* mutation. Previous studies have reported the role of *gidB* mutations in conferring a lower level of STR resistance [8]–[10]. The results of this study are in line with this observation, where the MIC_50_s for all *gidB* mutation-containing isolates and those without *rrs* or *rpsL* mutations were of 1.5 and 0.38 µg/ml, as opposed to 256 µg/ml for *gidB* mutation-free isolates but with alterations in *rrs* or *rpsL* genes. However, when analyzing the MICs for individual *gidB* mutants (without *rrs* or *rpsL* mutations), no consistency between the presence of a mutation type and MIC value was observed. Altogether, it seems that *gidB* mutations do play a role in STR resistance, as they occur in STR-resistant isolates with no additional mutations in other genes. However, the exact importance of these mutations for STR resistance and its level needs to be further investigated. Although the usefulness of *gidB* mutations as markers for STR resistance is rather doubtful or at least uncertain, they provide some interesting insights into the phylogeny of tubercle bacilli. The most striking finding is that 47T→G (L16R) mutation appears to be specific for the Latin American-Mediterranean (LAM) genotype family, whereas 276A→C (E92D) and 615A→G (A205A) polymorphisms seem to be specific for the Beijing genotype family [7], [20], [27], [29]. This relation was supported in the present study. All but two isolates belonging to the LAM genotype harbored the L16R mutation (the remaining two had a wild-type *gidB* sequence) (Table S2).

Considering the 4 Beijing genotype isolates, two, with an altered *gidB* allele, displayed a combination of the E92D and A205A mutations (Figure 2).

To better recognize the specificity of the *gidB* gene mutations detected for the LAM and Beijing families, they were searched against the whole genome sequences of *M. tuberculosis* strains, deposited in the GenBank database. Screening for the 47T→G (L16R) mutation was performed only on those strains which harbored 309G→A and 1212C→G polymorphisms in *Rv0129c* and *Rv3062* genes, respectively, suggested to be specific for the LAM family [36], [37]. Whereas, screening for the 276A→C (E92D) and 615A→G (A205A) mutations was done only for the strains that were positive for the 191A→C mutation in the *Rv2629* gene, a specific marker of the Beijing lineage [38]. All six *M. tuberculosis* strains from the GenBank database, confirmed to represent the LAM family, by the presence of mutations in *Rv0129c* and *Rv3062* genes, did contain the 47T→G mutation in the *gidB* gene. Likewise, all eight *M. tuberculosis* strains from the GenBank database, belonging to the Beijing family (mutation at *Rv2629*), did contain the 276A→C and 615A→G polymorphisms in the *gidB* gene.

Collectively, although the association of the *gidB* polymorphisms with specific lineages requires confirmation in broader population samples, the three polymorphisms described could be used as new phylogenetic markers to distinguish between different genogroups, some of which are of high epidemiological relevance. The Beijing and LAM genotypes are the most prominent, having repeatedly been associated with drug resistance, including MDR [39], [40]. Interestingly, isolates whose spoligotyping family was unknown had their STR MICs significantly lower than isolates of the LAM, Beijing, and Haarlem (H) genotypes (*P*≤0.04).

Noticeably, 12.5% (4/32) of the STR-resistant isolates had no mutations at any of the three *loci* investigated. This is much lower than what was observed previously in Poland (51%) [21], in Spain (62.3%) [6], Mexico (52%) [26], or Portugal (33.3%) [33]. An explanation for this difference may lie in the fact that all studies cited above did not perform *gidB* gene mutation profiling. In those few studies that involved the three-gene panel (*rrs*, *rpsL*, and *gidB*), the proportions of STR-resistant isolates with wild-type alleles at all three *loci* were similar to that from this study, being 6.9% in Viet Nam [7], 11.8% in Korea [19], 12.5% in Sierra Leone [27], and 22% in Brazil [29]. From these data, it is clear that mechanisms other than mutations in the *rrs*, *rpsL*, or *gidB* genes must exist to mediate STR resistance. One hypothesized mechanism includes changes in the cell envelope leading to decreased permeability, reduced uptake or increased efflux of the drug [4]. Further research are required to confirm this mechanism or identify additional mechanisms conferring STR resistance in *M. tuberculosis*.

In conclusion, this study adds importantly to the still limited body of evidence on the complexity of the genetic background of STR resistance in TB. The major findings from this study can be summarized in four points. Firstly, all non-synonymous mutations at either *rrs* or *rpsL* gene were found exclusively in STR-resistant isolates, and accounted for 71.9% (23/32) of them, supporting the suitability of *rrs* and *rpsL* mutations as predictive markers for STR resistance. Secondly, the *gidB* gene was highly polymorphic, with most of the polymorphisms having occurred in both STR-resistant and -susceptible isolates. This implies the role of *gidB* mutations as phylogenetic markers rather than markers for STR resistance. Indeed, the *gidB* polymorphisms at codons 16 and 92 or 205 were found to be associated with the LAM and Beijing genotype families, respectively. Thirdly, none of the mutations in any of the three *loci* evaluated could be conclusively linked to low, intermediate or high level of STR resistance. It seems that factors other than genetic alterations, including environmental determinants, may have an impact on the MIC values. Finally, finding of four (12.5%) STR-resistant isolates without any mutation in the three *loci* examined suggests the existence of other mechanisms involved in the development of STR resistance in tubercle bacilli.

## Supporting Information

Table S1 Distribution of *rrs*, *rpsL*, and *gidB* mutations among SM-resistant (*n* = 32) and SM-susceptible (*n* = 32) *M. tuberculosis* isolates, with regard to MIC values of SM.
(DOCX)

Table S2 Spoligotypes, resistance profiles and mutational patterns at *rrs*, *rpsL*, and *gidB* genes of the 64 *M. tuberculosis* isolates studied.
(XLSX)
