# Supplementary material for: Screening for Streptomycin Resistance-Conferring Mutations in Mycobacterium tuberculosis Clinical Isolates from Poland
Source: PLoS One. 2014 Jun 17;9(6):e100078. doi: 10.1371/journal.pone.0100078 (PMC4061058; doi:10.1371/journal.pone.0100078)
Supplement: Table S1 — Distribution of rrs, rpsL, and gidB mutations among SM-resistant (n = 32) and SM-susceptible (n = 32) M. tuberculosis isolates, with regard to MIC values of SM. (DOCX) [file pone.0100078.s001.docx]

**TABLE S1**. Distribution of *rrs*, *rpsL*, and *gidB* mutations among SM-resistant (*n*=32) and SM-susceptible (*n*=32) *M. tuberculosis* isolates, with regard to MIC values of SM

|  | Locus | Mutation*^a^* | | No. of isolates inhibited by a MIC [µg/ml] of: | | | | | | | | | | | | | | | | MIC_50_ | MIC_90_ | Range | GM*^b^* |
| --- | --- | --- | --- | --- | --- | --- | --- | --- | --- | --- | --- | --- | --- | --- | --- | --- | --- | --- | --- | --- | --- | --- | --- |
|  |  | NT | AA | <0.064 | 0.094 | 0.125 | 0.25 | 0.38 | 0.75 | 1.5 | 2 | 3 | 64 | 96 | 128 | 256 | 512 | 768 | >1024 | [µg/ml] | | | |
|  | *rrs* | | | | | | | | | | | | | | | | | | | 256 | >1024 | 0.25- >1024 | 84.2 |
| 1. |  | 514A→C | NA |  |  |  |  |  |  |  |  |  |  |  |  | 1 |  |  |  |  |  |  |  |
| 2. |  | 517C→T | NA |  |  |  | 1 |  |  |  |  |  |  | 1 |  |  |  |  | 2 |  |  |  |  |
| 3 |  | 906A→G^*^ | NA |  |  |  |  |  |  |  |  |  |  |  |  |  |  |  | 1 |  |  |  |  |
| 4. |  | 907A→T | NA |  |  |  |  |  | 1 |  |  |  |  |  |  |  | 1 |  |  |  |  |  |  |
|  | *rpsL* | | | | | | | | | | | | | | | | | | | 256 | >1024 | 0.125->1024 | 100.7 |
| 1. |  | 117C→T | T39T |  |  | 1 |  | 1 |  |  |  |  |  |  |  |  | 1 |  |  |  |  |  |  |
| 2. |  | 128A→G | K43R |  |  |  |  |  |  |  |  | 1 | 1 |  | 2 | 2 | 2 |  | 6 |  |  |  |  |
| 3. |  | 263A→G | K88R |  |  |  |  |  | 1 |  |  |  |  |  |  |  |  |  |  |  |  |  |  |
|  | *gidB* | | | | | | | | | | | | | | | | | | | 1.5 | >1024 | <0.064->1024 | 6.3 |
| 1. |  | 40delC^*^ | P14fs |  |  |  |  |  |  | 1 |  |  |  |  |  |  |  |  |  |  |  |  |  |
| 2. |  | 47T→G | L16R | 2 | 1 |  | 1 |  |  |  |  | 1 |  |  | 1 |  | 2 | 1 | 2 |  |  |  |  |
| 3. |  | 60insC^*^ | R21fs |  |  |  |  |  |  |  |  |  |  |  |  |  | 1 |  |  |  |  |  |  |
| 4. |  | 62G→A^*^ | R21Q |  |  |  |  |  |  |  |  |  |  |  |  |  |  |  | 1 |  |  |  |  |
| 5. |  | 98delG | R33fs | 1 |  |  |  |  |  |  |  |  |  |  |  |  |  |  |  |  |  |  |  |
| 6. |  | 112delC | P38fs |  |  | 1 |  |  |  |  | 1 |  |  |  |  |  |  |  |  |  |  |  |  |
| 7. |  | A119G^*^ | E40G |  |  |  | 1 |  |  |  |  |  |  |  |  |  |  |  |  |  |  |  |  |
| 8. |  | 154delT^*^ | C52fs |  |  | 1 |  | 1 |  |  |  |  |  |  |  |  |  |  |  |  |  |  |  |
| 9. |  | 155G→A^*^ | C52Y |  |  | 1 |  | 1 |  |  |  |  |  |  |  |  |  |  |  |  |  |  |  |
| 10. |  | 258insA^*^ | Q87fs |  |  |  |  |  |  | 1 |  |  |  |  |  |  |  |  |  |  |  |  |  |
| 11. |  | 276A→C | E92D |  |  |  |  |  |  |  |  |  |  |  |  |  |  |  | 2 |  |  |  |  |
| 12. |  | 351-2delGC^*^ | G117fs |  |  |  |  | 1 |  |  |  |  |  |  |  |  |  |  | 1 |  |  |  |  |
| 13. |  | 483C→G | A161A | 1 |  |  |  |  |  |  |  |  |  |  |  |  |  |  |  |  |  |  |  |
| 14. |  | 523C→G^*^ | R175G |  |  | 1 |  | 1 |  |  |  |  |  |  |  |  |  |  |  |  |  |  |  |
| 15. |  | 559A→C^*^ | R187R | 1 |  |  |  |  |  |  |  |  |  |  |  |  |  |  |  |  |  |  |  |
| 16. |  | 615A→G | A205A |  |  |  |  |  |  |  |  |  |  |  |  |  |  |  | 2 |  |  |  |  |

*^a^* NT, nucleotide sequence; AA, amino acid sequence; NA, not applicable; del, deletion; ins, insertion; fs, frameshift mutation;

*^b^* GM, geometric mean;

The vertical line in the middle of the table represents the break-point for SM resistance (MIC>4 µg/ml). Numbers in squares designate isolates which were identified as SM-resistant using the proportion method. Asterisks (^*^) indicate novel mutations (not previously reported in the literature).
